# Sources of CNS tumor heterogeneity

**DOI:** 10.18632/oncoscience.60

**Published:** 2014-07-02

**Authors:** Fuyi Chen, Albert Becker, Joseph LoTurco

**Affiliations:** Department of Physiology and Neurobiology, University of Connecticut, Storrs, USA

Central nervous system tumors are marked with high intra- and inter-tumoral heterogeneity. For instance, an integrative large scale gene expression study performed in 2010 revealed 4 subtypes glioblastoma multiforme (GBM) with differential responses to treatment and prognosis [1]. A more comprehensive GBM classification based on combination of epigenetics, copy number variation, gene expression and genetic mutation analysis has led to identification of as many as 6 GBM subgroups [2].

Two, not mutually exclusive, general models have been proposed to explain tumor heterogeneity [3]. The genetic mutation model proposes that different genetic mutations lead to different tumor formation, while the cell of origin model explains different tumors as arising from different cell types. There is experimental evidence supporting both models. For example, 3 distinctly different CNS tumor types can be induced by infection of postnatal mouse neural stem cells with virus containing V12HRAS and c-MYC depending on the combination and sequence in which oncogenes are introduced [4]. Similarly, RNA interference (RNAi) knock down of NF1 and p53 in GFAP+ or SynI+ cells induces mesenchymal GBM, whereas the same RNAi in Nestin+ cells induced neural GBM[5]. GMB gene expression analysis also indicates that different GBM subtypes have transcipt profiles similar to different cell types [1].

To fully explore the causes of tumor diversity, it is desirable^2^ to have an animal model in which both the cell of origin and genetic insult can be conveniently and independently manipulated. To achieve this, we have recently developed a central nervous system tumor model in the rat in which multiple oncogenes can be expressed in selected cell populations at different times in brain development [6]. In this model, we used the *piggyBac* transposon system [7] to stably integrate oncogenes into defined cell populations by *in utero* electroporation (IUE). Using this model, we evaluated the contribution of cell of origin and genetic mutation in tumor heterogeneity.

To test whether the same oncogenic event in different, but closely related, cell population gives rise to same or different tumors, we directed HRasV12/AKT expression in disparate cell populations in the radial glia lineage with promoters that are ubiquitously active (CAG promoter), astrocyte selective GFAP (glial fibrillary acidic protein) promoter or oligodendrocytes selective MBP (myelin basic protein) promoter. We showed that HRasV12/AKT expression under CAG or GFAP promoter induced similar tumors, glioblastoma multiforme (WHO grade 4). However, HRasV12/AKT expression controlled by MBP promoter induced anaplastic oligoastrocytoma (WHO grade 3). We further showed that these induced induced anaplastic oligoastrocytoma differed from glioblastoma multiforme both in histology and molecular signature. These results indicate that oncogenic events occurring in different cell types in the same cellular lineage can lead to different tumor types.

We next investigated whether tumor phenotype could be modified by expression of neurogenic bHLH family protein Neurogenin2 (Ngn2) or Neural differentiation 1 (NeuroD1). Members of the bHLH gene family are well known to have important roles is cell-type determination in normal development. Expression of either Ngn2 or NeuroD1 along with HRasV12/AKT resulted in atypical teratoid rhabdoid tumor like (ATRT like) tumor, a tumor type not previously observed after expression of HRasV12/AKT oncogenes alone. We further tested whether this phenotypic transformation from GBM to ATRT like tumor was due to transient expression of bHLH factors in radial glia or due to expression in tumor cells. Our data showed that transient expression of Ngn2 in radial glia, prior to transformation by HRasV12/AKT was able to induce ATRT like tumor formation. These results may indicate that the same oncogenic events occurring in similar cell types expressing different levels of individual bHLH transcription factors can lead to very different tumor types.

The main advantage of *piggyBac* IUE method is that it allows for introduction of multiple transgenes controlled by independent promoters. The high co-expression rates allowed us to direct expression in different subpopulations in sequence. In the same system, we also demonstrated use of multi-color fluorescent protein expression to produce a clonal readout of tumor growth and invasion. There are several additional features of the *piggyBac* system that make it useful for other applications in investigating tumor biology. For example, the *piggyBac*- IUE approach could be applied to other species other than rodent extending CNS tumor biology to other species, such as ferret. Combination of *piggyBac*-IUE with existing transgenic mouse models could potentially broaden the utility of both approaches. Also multicolor clonal labeling of tumor cells could facilitate *in vivo* imaging of clonally related tumor cells. These functionalities should make this approach a useful platform for screening potential modifiers of tumor development and for studying further how genetic modifiers and cell or origin are related to tumor development and heterogeneity.
